# Atypical bilateral papilledema during the puerperium: a case report

**DOI:** 10.3389/fmed.2025.1636933

**Published:** 2025-07-04

**Authors:** Ligang Jiang, Xin Jiang, Ailian Li, Mengting Liu, Zhe Zhang, Yuhua Tong

**Affiliations:** ^1^Department of Ophthalmology, The Quzhou Affiliated Hospital of Wenzhou Medical University, Quzhou People’s Hospital, Quzhou, China; ^2^Quzhou College of Technology, Quzhou, China; ^3^Department of Ophthalmology, The Second Xiangya Hospital, Central South University, Hunan Clinical Research Centre of Ophthalmic Disease, Changsha, China; ^4^Shenzhen Eye Hospital, Shenzhen Eye Medical Center, Southern Medical University, Shenzhen, China

**Keywords:** puerperium, papilledema, high intracranial pressure, intracranial venous sinus stenosis, hypercoagulable state of pregnancy, abnormalities in immunity, case report

## Abstract

**Background:**

To analyze a case of atypical bilateral papilledema in a puerperium woman, and to explore the pathogenic mechanism of pregnancy-related physiological changes, blood hypercoagulable state, immune abnormalities and abnormal structure of intracranial venous sinus, so as to provide reference for early diagnosis and intervention of similar cases.

**Case report:**

A 28-year-old woman, 3 days post-operative from a cesarean section, presented at the hospital with decreased vision in her right eye. An examination revealed bilateral papilledema. She did not have typical symptoms like dizziness, headache, or pulsatile tinnitus. During pregnancy, she had taken hydroxychloroquine orally for 5 months due to elevated immune indexes. She also received anticoagulant therapy for lower extremity venous thrombosis a month prior and had a history of cerebrospinal fluid leakage repair for intracranial hypotension syndrome a year ago. Fundus photography and OCT showed bilateral papilledema and macular edema in the right eye, with slightly enlarged physiological blind spots in both eyes. Her pre-pregnancy BMI was 16.5, and postpartum BMI was 22. Laboratory tests indicated a D-dimer level exceeding 20 mg/L and abnormal immune indicators. Ophthalmic color Doppler ultrasound demonstrated bilateral optic nerve sheath widening, with measurements of 0.625 cm on the right and 0.590 cm on the left, suggesting potential elevated intracranial pressure. MRV detected stenosis in the right distal sigmoid sinus and proximal transverse sinus, while the left sigmoid sinus and transverse sinus were not visualized. The patient was diagnosed with increased intracranial pressure caused by multiple factors. Treatment with mannitol to reduce intracranial pressure, along with anticoagulation and other supportive and symptomatic treatments, was administered. After 1 week, macular edema in the right eye subsided, vision improved, and bilateral papilledema slowly improved.

**Conclusion:**

This case provides multi-dimensional clinical evidence for the differential diagnosis of puerperium papilledema. For patients with low BMI and atypical symptoms of bilateral papilledema during puerperium, it is necessary to be alert to multiple pathogenic factors. It is recommended to preferentially screen intracranial venous sinus lesions and detect immune indicators by imaging. Ocular ultrasound can be used as a non-invasive screening method for intracranial hypertension.

## Introduction

Papilledema, characterized by hyperemia, edema, and elevation of the optic nerve head, serves as a critical warning sign of central nervous system disorders (1). Bilateral involvement particularly often indicates etiologies secondary to intracranial hypertension, such as brain tumors, idiopathic intracranial hypertension (IIH), or intracranial venous sinus thrombosis (CVST) (2). In reproductive-aged women, IIH represents a common cause of bilateral papilledema, typically characterized by headache, pulsatile tinnitus, and a strong association with elevated body mass index (BMI) (3, 4). However, due to pregnancy-related physiological changes, the pathogenesis of papilledema in puerperal women may involve unique mechanisms. This requires vigilance for synergistic contributions from multifactorial influences beyond traditional causes, including non-intracranial hypertension etiologies such as optic perineuritis, incipient non-arteritic anterior ischemic optic neuropathy, hypertensive emergency, and intermediate uveitis (5).

However, there are few reports on puerperal papilledema cases of “low BMI women with a history of intracranial hypotension.” This study reports a case of bilateral papilledema in a 28-year-old low BMI woman after cesarean section. The purpose of this study is to explore the synergistic mechanism of pregnant-related physiological changes, hypercoagulable state, immune abnormalities and structural abnormalities of intracranial venous sinus, so as to provide a new perspective for the early differential diagnosis of puerperal papilledema, especially in patients with low BMI and atypical symptoms.

## Case presentation

**Figure 1:**
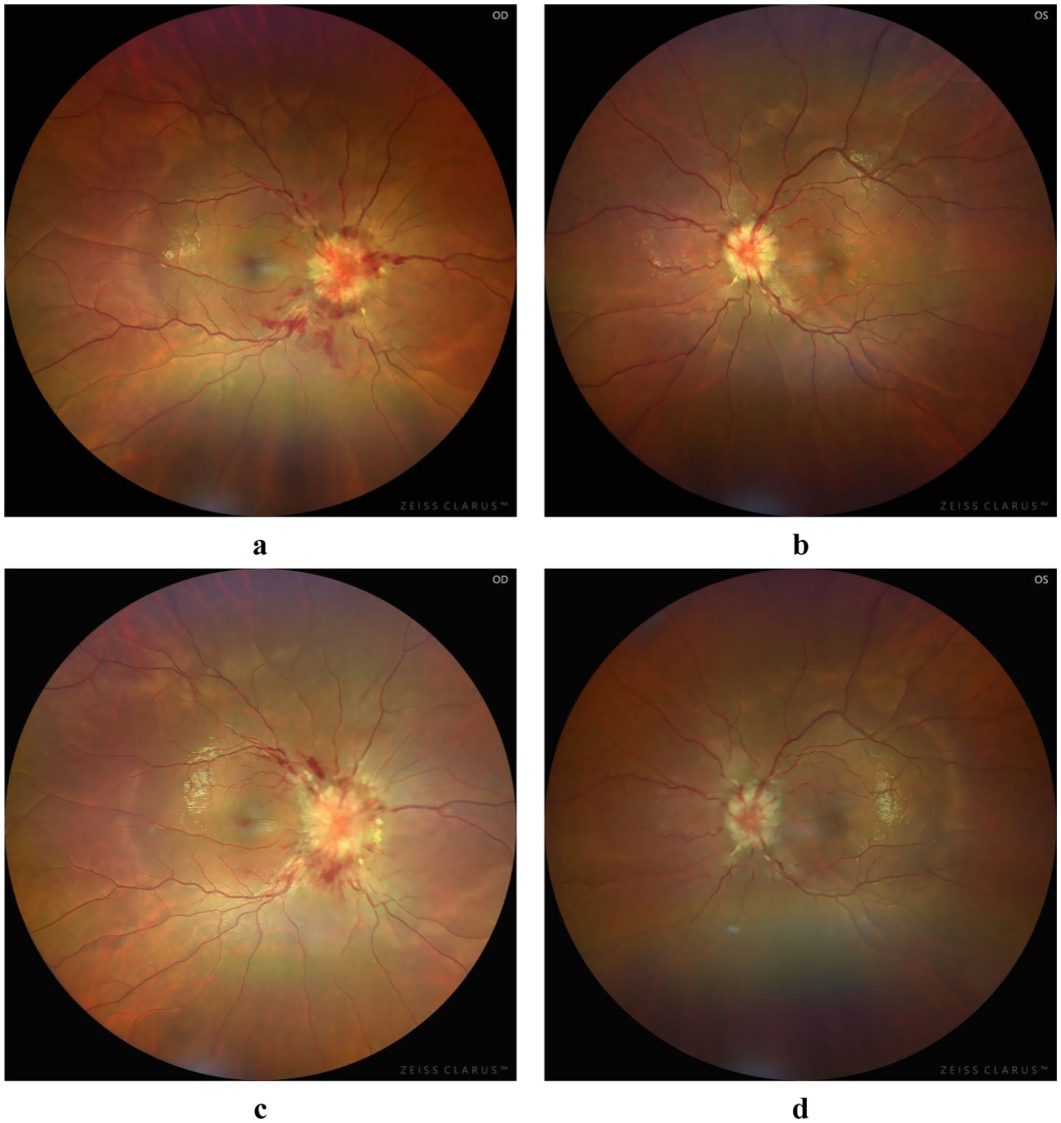
Fundus photograph: at initial diagnosis, significant bilateral papilledema was observed, characterized by blurred margins, tortuous and dilated peripapillary veins, and patchy hemorrhage **(a,b)**. Following treatment, the tortuosity and dilatation of the bilateral peripapillary veins improved, the hemorrhage resolved, and the papilledema gradually improved **(c,d)**.

**Figure 2:**
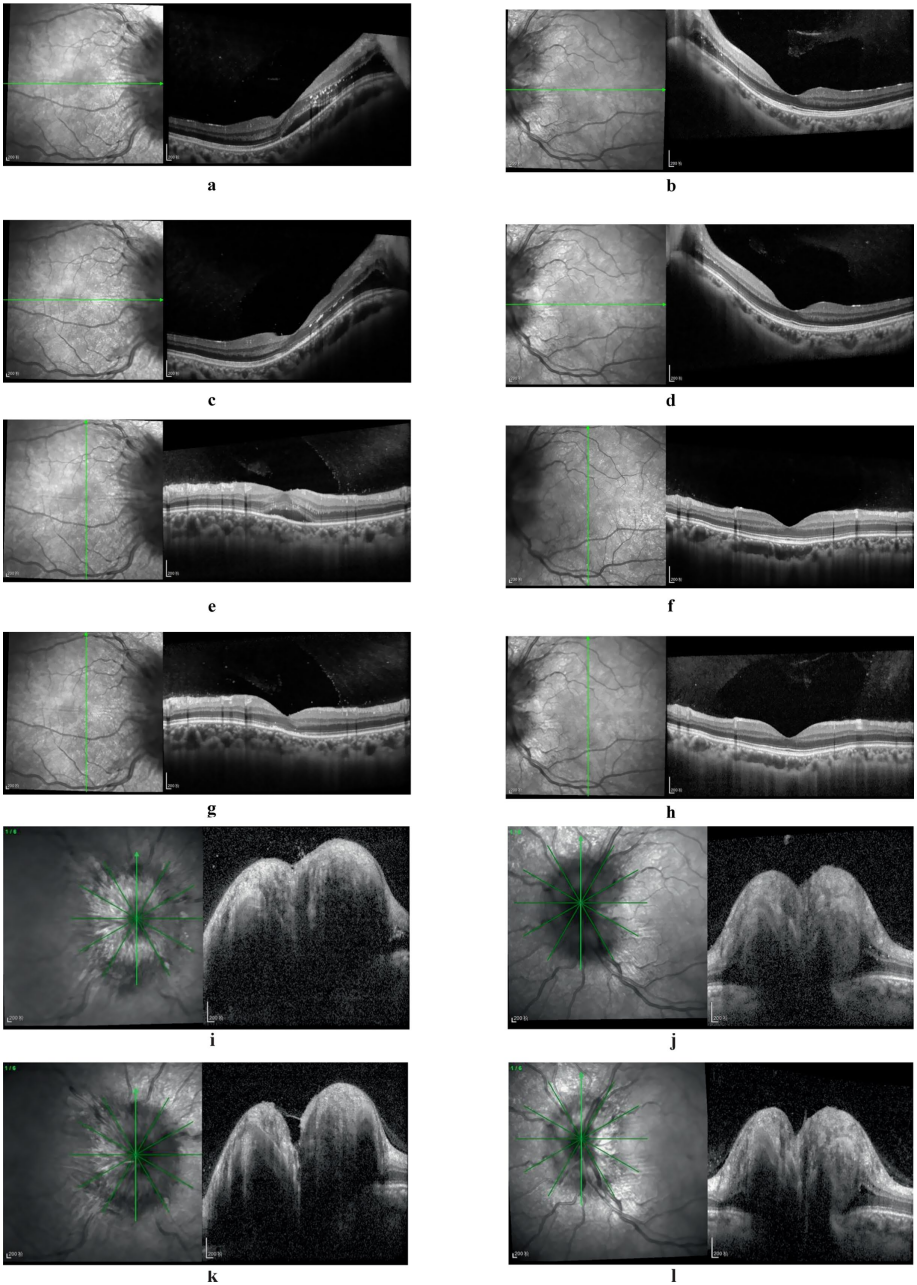
Fundus OCT images: at initial diagnosis, partial edema extending from the macular region to the optic nerve head was observed in the right eye, accompanied by minor exudation and retinal thickening **(a,e)**. Post-treatment, macular edema resolved, exudation progressively diminished, retinal thickness normalized, yet the ellipsoid zone exhibited partial absence and fragmentation **(c,g)**. The left eye’s macular region displayed no significant abnormalities **(b,d,f,h)**. Bilateral papilledema was evident **(i,j)**, with slight resolution following treatment **(k,l)**.

**Figure 3:**
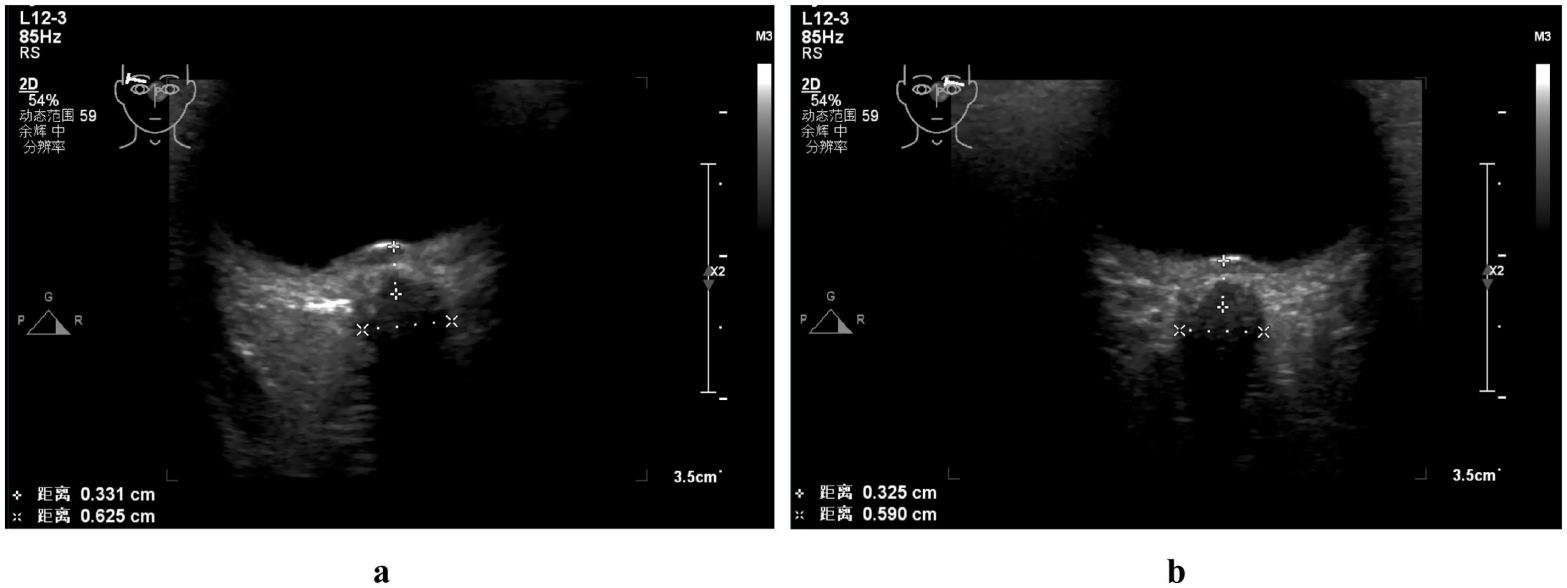
Color Doppler ultrasonographic examination of the bilateral optic nerve sheath: the optic nerve sheath diameter was measured at approximately 0.625 cm on the right side **(a)** and 0.590 cm on the left side **(b)**.

**Figure 4:**
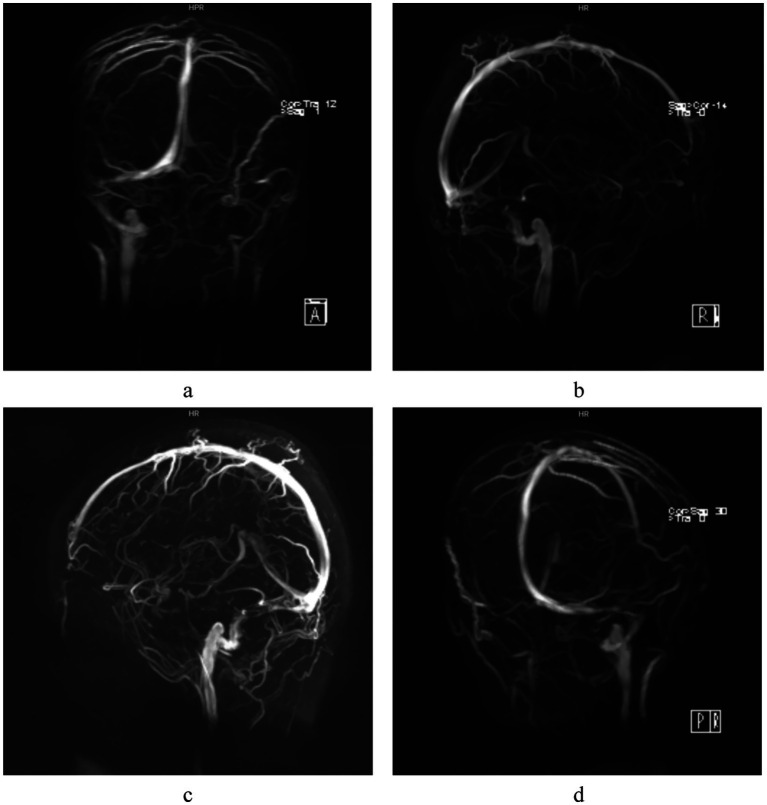
MRV examination: Focal narrowing of the right distal sigmoid sinus and proximal transverse sinus is demonstrated in different angles **(a-d)**, no imaging of the left sigmoid sinus and transverse sinus is visualized.

The patient was a 28-year-old female who presented with decreased vision in the right eye postpartum. Fundus examination revealed bilateral papilledema. She reported no redness or eye pain, no dizziness, headache, nausea, vomiting, or pulsatile tinnitus. Due to elevated immune indicators during pregnancy, she had been taking hydroxychloroquine orally for over 5 months, which was discontinued a week prior. One month ago, the patient was diagnosed with lower extremity venous thrombosis accompanied by elevated D-dimer levels. She received systemic anticoagulation therapy with subcutaneous injection of low molecular weight heparin at a dose of 200 IU/kg administered once daily. This therapy was continued until the day prior to delivery. Post-delivery, the patient’s anticoagulant regimen was switched to oral rivaroxaban tablets at a dosage of 10 mg daily. A cesarean section was performed 3 days prior. Her pre-pregnancy weight was 45 kg (BMI 16.5), and postpartum weight was 60 kg (BMI 22). Her blood pressure remained normal, with no history of gestational hypertension or diabetes. One year ago, she was diagnosed with intracranial hypotension syndrome due to “headache” and underwent cerebrospinal fluid leakage repair. There was no significant family or other medical history. Physical examination showed normal muscle tone and strength in the extremities, and normal deep and superficial sensation. Uncorrected visual acuity was 0.6 in the right eye and 1.0 in the left. Slit-lamp examination of the anterior eye segments revealed no abnormalities but showed vitreous opacity and posterior detachment. The physiological blind spots in both eyes’ visual fields were enlarged. Fundus photography (Figure 1) showed prominent papilledema with blurred edges, tortuous and dilated peripheral veins, and patchy bleeding in both eyes. OCT (Figure 2) revealed partial edema from the macular to the optic-disk area in the right eye, with minor exudation and retinal thickening. The left eye’s macular area showed no significant abnormalities. Blood tests indicated D-dimer >20 mg/L, erythrocyte sedimentation rate 78 mm/h, antinuclear antibody 333.10 AU/mL, weakly positive anti-SSA/Ro52 and anti-SSA/Ro60 antibodies, complement C3 1.81 g/L, and C4 0.83 g/L, anti-double-stranded DNA antibodies were negative, the lupus screening ratio was normal, anticardiolipin antibodies were normal, and no other blood tests were significantly abnormal. To rule out other secondary causes of increased intracranial pressure, an MRI was performed, but it revealed no significant abnormalities. We advised the patient to undergo lumbar puncture to measure intracranial pressure. However, due to her puerperal status and previous history of low intracranial pressure, she was concerned about the risk of lumbar puncture-induced cerebrospinal fluid leakage and disagreed with the high intracranial pressure diagnosis. Ophthalmic color Doppler ultrasound demonstrated bilateral optic nerve sheath widening, with measurements of 0.625 cm on the right and 0.590 cm on the left (Figure 3), suggesting potential elevated intracranial pressure. Lower extremity venous color Doppler ultrasound revealed thrombosis in one of the left posterior tibial veins. MRV examination (Figure 4) showed focal thinning of the right distal sigmoid sinus and proximal transverse sinus, non-visualization of the left sigmoid sinus and transverse sinus, and thinning of the left distal internal jugular vein. We concluded that the patient’s increased intracranial pressure, leading to bilateral papilledema, was caused by a combination of pregnancy-related physiological changes, blood hypercoagulability, immune abnormalities, and intracranial venous sinus structural anomalies. Treatment with mannitol to reduce intracranial pressure, along with microcirculation improvement, neurotrophic therapy, and systemic anticoagulation, was administered. After 1 week, visual acuity improved, with uncorrected visual acuity reaching 1.0 in both eyes. Fundus reexamination showed alleviated tortuous dilation of the bilateral peripapillary veins, reduced and absorbed bleeding, gradually decreased exudation, and slowly improving papilledema (Figures 1, 2). In the right eye, macular edema resolved, exudation gradually decreased, retinal thickness returned to normal, and the ellipsoid zone was partially absent and broken (Figure 2). The left eye’s macular area showed no apparent abnormalities (Figure 2), and the patient continues to receive further treatment.

## Discussion

There are two key factors contributing to the difficulty in making a definite diagnosis in this case. From the perspective of intracranial pressure evaluation, the clinical symptoms of high intracranial pressure were not significant. Moreover, the patient had undergone cerebrospinal fluid leak repair surgery 1 year prior, making it challenging to attribute the current clinical signs solely to intracranial hypertension. Additionally, the patient refused lumbar puncture examination, leaving the exact value of intracranial pressure uncertain. Considering the systemic condition, the patient’s condition was complex, and the presence of systemic underlying diseases along with the interaction of multiple factors further increased the diagnostic difficulty.

In terms of differential diagnosis, given the abnormal systemic immune indicators during pregnancy and the long-term oral administration of hydroxychloroquine, systemic immune diseases such as systemic lupus erythematosus (SLE) should be distinguished. As a chronic autoimmune disease with diverse clinical manifestations, SLE can involve the posterior segment of the eye. Its ocular lesions were once thought to be related to idiopathic intracranial hypertension (6). Taba et al. (7) reported a rare case of a young female SLE patient who presented with bilateral papilledema and macular edema, despite normal lumbar puncture results and the exclusion of idiopathic intracranial hypertension. This suggests that SLE may have a mechanism of posterior ocular segment damage independent of intracranial hypertension.

Pregnancy induces physiological changes that can affect various body systems, including the cardiovascular and endocrine systems, potentially leading to complications like gestational diabetes and eclampsia (8–10). However, whether these hormonal changes also cause biochemical, morphological, or functional changes in the central nervous system remains unclear. Recent studies suggest that pregnancy-related hormonal fluctuations can interact with the central nervous system, causing neuronal enlargement and subsequent abnormal cerebrospinal fluid (CSF) pressure (11). Additionally, pregnancy-associated factors such as decreased plasma osmolality and albumin concentration, increased blood volume and cardiac output, and sodium and free water retention can predispose women to cerebral edema (12), potentially increasing intracranial pressure and causing papilledema. IIH primarily affects individuals with a high BMI in their childbearing years (13), with hormonal changes and rapid weight gain possibly acting as triggers for IIH. Thus, pregnancy may represent a potential risk factor for IIH (14, 15).

Autoimmune diseases are important risk factors for CVST (16, 17). Autoimmune inflammatory activation may contribute to intracranial venous sinus inflammation via vascular endothelial injury. Thrombotic events occur in 10–20% of SLE patients (18), resulting from lupus anticoagulant deposition combined with immune-mediated vasculitis (19). Elevated antinuclear antibodies, weakly positive anti-SSA antibodies, and increased C3 and C4 levels may play roles in pregnancy-related cerebral venous sinus thrombosis. Pregnancy and puerperium are the most common risk factors for CVST (20, 21). The hypercoagulable state of blood and venous stasis, along with a significant increase in fibrinogen and coagulation factors, and a significant decrease in antithrombin III and plasminogen, increase the risk of CVST (22). Based on the patient’s comprehensive clinical data, including postpartum hypercoagulable state, venous stasis, D-dimer level >20 mg/L, previous history of lower extremity venous thrombosis, abnormal immune biomarkers, and MRV revealing focal stenosis of the distal right sigmoid sinus and proximal transverse sinus, agenesis of the left sigmoid sinus and transverse sinus, and stenosis of the distal internal jugular vein, collectively these findings suggest the presence of occult microembolic obstruction and mild venous luminal narrowing. These may reduce blood flow in the right transverse and sigmoid sinuses. The underdevelopment of the left venous sinus could be due to complete thrombotic occlusion or congenital factors, as the left lateral sinus is typically hypoplastic. Jianu et al. (23) observed that IIH often occurs following thrombosis of the right lateral sinus. Arias-Moeller et al. (24) demonstrated that patients with CVST complicated by contralateral dysplastic venous sinuses have a significantly higher risk of developing intracranial hypertension. The pathogenesis of intracranial hypertension in this patient is further complicated by a prior history of intracranial hypotension repair surgery, which inherently adds diagnostic complexity and clinical challenges. CVST frequently occurs as a secondary consequence of intracranial venous sinus stenosis (25), with the latter serving as a key predisposing factor for the former. Both intracranial venous sinus thrombosis and stenosis can impair venous return, thereby increasing venous sinus pressure (26). This pressure elevation disrupts CSF resorption mechanisms, leading to CSF accumulation and a subsequent gradual increase in intracranial pressure (27). The resulting pressure imbalance within the optic nerve sheath promotes papilledema formation, ultimately establishing a vicious cycle of “stenosis-pressure-sinus wall collapse-stenosis exacerbation.” The causal relationship between venous sinus stenosis and increased intracranial pressure remains unclear (28). Rohr et al. (29) suggested that venous sinus stenosis impedes CSF reflux, affecting CSF absorption by arachnoid granulations and elevating intracranial pressure. Conversely, Puffer et al. (30, 31) posited that increased intracranial pressure compresses the venous sinuses, reducing their compliance and causing sinus wall collapse. Regardless of whether venous sinus stenosis is the cause or consequence of IIH, venous sinus hypertension is a key factor in exacerbating cerebral circulatory disorders (32). Although lumbar puncture was not performed herein, ophthalmic color Doppler ultrasound showed bilateral optic nerve sheath widening, about 0.625 cm on the right and 0.590 cm on the left, which supported the diagnosis of intracranial hypertension. Multiple studies (33–36) have established ocular ultrasound as a reliable non-invasive diagnostic tool for assessing intracranial pressure changes. It can effectively evaluate intracranial pressure via the optic nerve sheath diameter with good sensitivity, offering a safer and more effective alternative to invasive lumbar puncture. The limitation is that ophthalmic color Doppler ultrasound is strongly operator-dependent. Cimilli Ozturk et al. (37) showed that there were differences in measurements between operators, which means that examination results may vary depending on the experience and skill of the operator.

In conclusion, the present case may be caused by multiple factors, such as pregnancy-related physiological changes, a hypercoagulable state, immune abnormalities, and abnormal intracranial venous sinus structure. The sustained increase in estrogen and progesterone levels during pregnancy can increase vascular endothelial permeability, potentially elevating postpartum intracranial pressure. Although the elevated antinuclear antibody levels and complement system activation do not meet the diagnostic criteria for autoimmune diseases, they create a chronic vascular endothelial inflammatory microenvironment. This promotes the activation of coagulation factors and inhibits the anticoagulant system, creating an “additive effect” that exacerbates the pregnancy-related hypercoagulable state. The patient’s history of lower extremity venous thrombosis further supports the presence of systemic hypercoagulability.

## Conclusion

Papilledema is a typical manifestation of optic nerve head injury, particularly in bilateral cases. The direct cause of papilledema is increased intracranial pressure, and its root cause may stem from central nervous system diseases, systemic immune system diseases, pregnancy-related physiological changes, and other factors. Therefore, comprehensive and systematic differential diagnosis and clinical analysis are of key clinical significance for accurately identifying potential causes and avoiding the risk of misdiagnosis (38).

## Data Availability

The original contributions presented in the study are included in the article/Supplementary material, further inquiries can be directed to the corresponding authors.

## References

[1] Abbas M Alahmad A Hamzeh G Haddeh Y. Bilateral swollen optic nerve head etiology and management: a cross-sectional study. Ann Med Surg. (2022) 79:104059. doi: 10.1016/j.amsu.2022.104059, PMID: 35860086 PMC9289387

[2] Xie JS Donaldson L Margolin E. Papilledema: a review of etiology, pathophysiology, diagnosis, and management. Surv Ophthalmol. (2021) 67:1135–59. doi: 10.1016/j.survophthal.2021.11.007 34813854

[3] Crum OM Kilgore KP Sharma R Lee MS Spiegel MR McClelland CM. Etiology of papilledema in patients in the eye clinic setting. JAMA Netw Open. (2020) 3:e206625. doi: 10.1001/jamanetworkopen.2020.6625, PMID: 32484553 PMC7267843

[4] Daniels AB Liu GT Volpe NJ Galetta SL Moster ML Newman NJ. Profiles of obesity, weight gain, and quality of life in idiopathic intracranial hypertension (pseudotumor cerebri). Am J Ophthalmol. (2007) 143:635–641.e1. doi: 10.1016/j.ajo.2006.12.040, PMID: 17386271

[5] Yu CW Micieli JA. Bilateral optic disc edema with preserved visual function not related to papilledema. J Neurol Sci. (2020) 418:117160. doi: 10.1016/j.jns.2020.117160, PMID: 33010652

[6] Zou M Jiang X Chen H Yuan F. Systemic lupus erythematosus with chronic persistent intracranial hypertension: a case report. Lupus. (2024) 33:293–7. doi: 10.1177/09612033241230734, PMID: 38285490

[7] Taba JAP Triningrat AAMP Wijayati MP Kusumadjaja IMA. Ocular involvement as the primary presentation of suspected systemic lupus erythematosus: a case of bilateral papilledema and macular edema. Biosci Med J Biomed Transl Res. (2025) 9:7664–76. doi: 10.37275/bsm.v9i6.1303

[8] Stuart JJ Tanz LJ Missmer SA Rimm EB Spiegelman D James-Todd TM. Hypertensive disorders of pregnancy and maternal cardiovascular disease risk factor development: an observational cohort study. Ann Intern Med. (2018) 169:224–32. doi: 10.7326/M17-2740, PMID: 29971437 PMC6601621

[9] Jiang L Ji Y Liu M Fang R Zhu Z Zhang M. Exploring the effect of gestational diabetes mellitus on retinal vascular morphology by PKSEA-Net. Front Cell Dev Biol. (2025) 12:1532939. doi: 10.3389/fcell.2024.1532939 39845084 PMC11750853

[10] Calina D Docea AO Golokhvast KS Sifakis S Tsatsakis A Makrigiannakis A. Management of endocrinopathies in pregnancy: a review of current evidence. Int J Environ Res Public Health. (2019) 16:781. doi: 10.3390/ijerph16050781, PMID: 30836653 PMC6427139

[11] Mafrici M Tona F Fragiotta S Lorenzi U Gitto L Toscani L. Idiopathic intracranial hypertension papillopathy due to hormonal changes during pregnancy. Case Rep Ophthalmol Med. (2023) 2023:6688445. doi: 10.1155/2023/6688445, PMID: 37469477 PMC10353893

[12] Quick AM Cipolla MJ. Pregnancy-induced up-regulation of aquaporin-4 protein in brain and its role in eclampsia. FASEB J. (2005) 19:170–5. doi: 10.1096/fj.04-1901hyp, PMID: 15677340

[13] Wardman JH Jensen MN Andreassen SN Styrishave B Wilhjelm JE Sinclair AJ. Modelling idiopathic intracranial hypertension in rats: contributions of high fat diet and testosterone to intracranial pressure and cerebrospinal fluid production. Fluids Barriers CNS. (2023) 20:44. doi: 10.1186/s12987-023-00436-1, PMID: 37328884 PMC10276479

[14] Palermo M Trevisi G D'Arrigo S Sturiale CL. Idiopathic intracranial hypertension in pregnancy. A systematic review on clinical course, treatments, delivery and maternal-fetal outcome. Eur J Neurol. (2025) 32:e70186. doi: 10.1111/ene.70186, PMID: 40391885 PMC12090364

[15] Thaller M Homer V Mollan SP Sinclair AJ. Disease course and long-term outcomes in pregnant women with idiopathic intracranial hypertension: the IIH prospective maternal health study. Neurology. (2023) 100:e1598–610. doi: 10.1212/WNL.0000000000206854, PMID: 36750388 PMC10103118

[16] Barber MRW Clarke AE Adams CD Skeith L. Severe thrombotic complications secondary to antiphospholipid syndrome and undiagnosed systemic lupus erythematosus. Can Med Assoc J. (2022) 194:E1243–7. doi: 10.1503/cmaj.220491, PMID: 36122922 PMC9484616

[17] Chen X Song S Zhang Z Wang J. Thrombotic diathesis combined with connective tissue disease in a Chinese teenage male: a case report. Int J Rheum Dis. (2025) 28:e70228. doi: 10.1111/1756-185X.70228, PMID: 40269468

[18] Flusser D Abu-Shakra M Baumgarten-Kleiner A Flusser G Sukenik S. Superior sagittal sinus thrombosis in a patient with systemic lupus erythematosus. Lupus. (1996) 5:334–6. doi: 10.1177/096120339600500416, PMID: 8869908

[19] Zhang B Lang Y Zhang W Cui L Deng F. Characteristics and management of autoimmune disease-associated cerebral venous sinus thrombosis. Front Immunol. (2021) 12:671101. doi: 10.3389/fimmu.2021.671101, PMID: 34367137 PMC8339549

[20] Akinshina S Bitsadze V Khizroeva J Tretyakova M Grigoreva K Gashimova N. Cerebral vein thrombosis: management tactics with a focus on pregnancy, the use of hormone therapy and assisted reproductive technologies. J Matern Fetal Neonatal Med. (2025) 38:2447349. doi: 10.1080/14767058.2024.2447349, PMID: 39757006

[21] Merz LE Bassa B Ní Áinle F Fogerty AE. Thrombotic complications in pregnancy: a case-based review of the evidence. J Thromb Haemost. (2025) 23:417–28. doi: 10.1016/j.jtha.2024.09.029, PMID: 39395543

[22] Bagot CN Leishman E Onyiaodike CC Jordan F Gibson VB Freeman DJ. Changes in laboratory markers of thrombotic risk early in the first trimester of pregnancy may be linked to an increase in estradiol and progesterone. Thromb Res. (2019) 178:47. doi: 10.1016/j.thromres.2019.03.015, PMID: 30965151

[23] Jianu DC Jianu SN Dan TF Munteanu G Copil A Birdac CD. An integrated approach on the diagnosis of cerebral veins and dural sinuses thrombosis (a narrative review). Life. (2022) 12. doi: 10.3390/life12050717, PMID: 35629384 PMC9145675

[24] Farias-Moeller R Avery R Diab Y Carpenter J Murnick J. Contralateral hypoplastic venous draining sinuses are associated with elevated intracranial pressure in unilateral cerebral sinovenous thrombosis. AJNR Am J Neuroradiol. (2016) 37:2392–5. doi: 10.3174/ajnr.A4899, PMID: 27469210 PMC7963867

[25] Zhao T Wang G Dai J Liu Y Wang Y Li S. Cases of visual impairment caused by cerebral venous sinus occlusion-induced intracranial hypertension in the absence of headache. BMC Neurol. (2018) 18:159. doi: 10.1186/s12883-018-1156-7, PMID: 30268100 PMC6162896

[26] Ming S Qi Z Wang L Zhu K. Deep cerebral venous thrombosis in adults. Chin Med J. (2002) 115:395–7. PMID: 11940373

[27] Zhao K Gu W Liu C Kong D Zheng C Chen W. Advances in the understanding of the complex role of venous sinus stenosis in idiopathic intracranial hypertension. J Magn Reson Imaging. (2022) 56:645–54. doi: 10.1002/jmri.28177, PMID: 35357056 PMC9541264

[28] Jiang L Liu M Yu M Lu W Zhang Z Tong Y. Application of the full-width-at-half-maximum image segmentation method to analyse retinal vascular changes in patients with internal carotid artery stenosis. Front Cell Dev Biol. (2024) 12:1467374. doi: 10.3389/fcell.2024.1467374 39224436 PMC11366705

[29] Rohr A Bindeballe J Riedel C van Baalen A Bartsch T Doerner L. The entire dural sinus tree is compressed in patients with idiopathic intracranial hypertension: a longitudinal, volumetric magnetic resonance imaging study. Neuroradiology. (2011) 54:25–33. doi: 10.1007/s00234-011-0850-6, PMID: 21340576

[30] Puffer RC Mustafa W Lanzino G. Venous sinus stenting for idiopathic intracranial hypertension: a review of the literature. J Neurointerv Surg. (2012) 5:483–6. doi: 10.1136/neurintsurg-2012-010468, PMID: 22863980

[31] Donnet A. Idiopathic intracranial hypertension: stent or not. Rev Neurol. (2012) 168:685–90. doi: 10.1016/j.neurol.2012.07.014, PMID: 22981295

[32] Dinkin M Oliveira C. Men are from Mars, idiopathic intracranial hypertension is from venous: the role of venous sinus stenosis and stenting in idiopathic intracranial hypertension. Semin Neurol. (2019) 39:692–703. doi: 10.1055/s-0039-3399506, PMID: 31847040

[33] De Bernardo M Vitiello L De Luca M La Marca A Rosa N. Optic nerve changes detected with ocular ultrasonography during different surgical procedures: a narrative review. J Clin Med. (2022) 11:5467. doi: 10.3390/jcm11185467, PMID: 36143114 PMC9500847

[34] Robba C Cardim D Tajsic T Pietersen J Bulman M Donnelly J. Ultrasound non-invasive measurement of intracranial pressure in neurointensive care: a prospective observational study. PLoS Med. (2017) 14:e1002356. doi: 10.1371/journal.pmed.1002356, PMID: 28742869 PMC5526499

[35] Lin JJ Chen AE Lin EE Hsia SH Chiang MC Lin KL. Point-of-care ultrasound of optic nerve sheath diameter to detect intracranial pressure in neurocritically ill children—a narrative review. Biomed J. (2020) 43:231–9. doi: 10.1016/j.bj.2020.04.006, PMID: 32335329 PMC7424084

[36] Wang P Zhou X Sheng F Wang X Shi C Feng W. Ultrasonic optic nerve sheath diameter can be used as a diagnostic measure after accidental dural puncture during cesarean section: a case report. BMC Anesthesiol. (2024) 24:35. doi: 10.1186/s12871-024-02418-8, PMID: 38254029 PMC10802025

[37] Cimilli Ozturk T Demir H Yorulmaz R Ozdemir S Isat G Ecmel Onur O. Assessment of intra-interobserver reliability of the sonographic optic nerve sheath diameter measurement. Kaohsiung J Med Sci. (2015) 31:432–6. doi: 10.1016/j.kjms.2015.06.004, PMID: 26228283 PMC11916907

[38] Zhang Q Zhang P Zhu S Gong D Wang S Xu Y. Research hotspots and trends of ophthalmic artificial intelligence based on WoSCC literature in the past 10 years. Digit Med Health. (2020) 2:240–6. doi: 10.3760/cma.J.c.n101909-20240424-00087

